# An eco-friendly method of extracting alizarin from *Rubia tinctorum* roots under supercritical carbon dioxide and its application to wool dyeing

**DOI:** 10.1038/s41598-022-27110-0

**Published:** 2023-01-02

**Authors:** Tarek Abou Elmaaty, Khaled Sayed-Ahmed, Mai Magdi, Hanan Elsisi

**Affiliations:** 1 Department of Textile Printing, Dyeing and Finishing, Faculty of Applied Arts, Damietta University, Damietta, 34512 Egypt; 2 Department of Agricultural Chemistry, Faculty of Agriculture, Damietta University, Damietta, 34512 Egypt

**Keywords:** Soft materials, Chemistry

## Abstract

Because of its low critical temperature and pressure levels, supercritical carbon dioxide (scCO_2_) is the most widely used supercritical fluid in the supercritical fluid extraction (SFE) technique. Alizarin was extracted from madder roots (*Rubia tinctorum*) using scCO_2_ under different conditions of co-solvent ratio (0–50%), temperature (45–95 °C), pressure (150–250 bar), extraction time (15–120 min), and flow rate (5–9 mL/min). Based on alizarin recovery and minimization of environmental risk, the optimum conditions were determined. SFE was optimum at 90% CO_2_:10% methanol (Me), 65 °C, 250 bar, 45 min, and 9 mL/min. The alizarin recovery, and its content in *R. tinctorum* extract (RE) under the optimum conditions were 1.34 g/kg roots, and 6.42%, respectively. Using conventional dyeing methods, wool fabrics were dyed with RE at different concentrations (2–6%). Various types of mordants were also used in the dyeing process, including chemical and bio-mordants. Color and fastness properties of dyed wool fabrics were evaluated based on RE concentration and mordant type. A higher RE concentration and the use of mordants, specifically *Punica granatum* (*P. granatum*) peels, increased the color characteristics. RE and dyed fabrics exhibited good antibacterial activity against the tested bacterial strains, especially *Pseudomonas aeruginosa* and *Escherichia coli*.

## Introduction

Eco-friendly approaches in the textile industry can be influenced by strict environmental regulations for textiles and garments set by countries concerned with the environment and human health^1,2^. Because of its wide range of functional properties and environmental benefits, sustainable dyeing with natural colorants has gained attention^3,4^. Synthetic dyes are toxic and cause allergic reactions; therefore, natural dyes have been chosen as sustainable sources of colorants^5,6^. Some natural dyes exhibit color performance comparable to that of some highly rated synthetic dyes in both natural and synthetic fabrics^7–10^. Anthraquinone, anthocyanin, flavonoid dyes, and polyphenolic compounds are the main components of natural dyes, which have a red shade that is relatively stable and light fast. In terms of natural dyes based on anthraquinones, *R. tinctorum* (madder) is one of the oldest. *R. tinctorum* is a perennial herbaceous plant that belongs to the Rubiaceae family. In Central Asia, Iran, and Egypt, it has been widely cultivated since 1500 BC. As a natural source of red, pink, orange, purple, gray, and brown, as well as the most valuable black and brunette shades with high color depth, *R. tinctorum* is widely regarded as the most popular^11–16^. In traditional extraction procedures, organic solvents may cause thermal degradation, oxidative changes in the components, and the generation of unacceptable residues or solvent traces^17,18^. These concerns have led to the development of innovative environmentally friendly processes using renewable and non-toxic solvents in response to environmental constraints, extremely strict public health legislation, and market trends toward ecology^19^. Natural dyes can be extracted using scCO_2_^20^. As an alternative to conventional extraction methods, supercritical fluid extraction follows green chemistry principles^21^.

Under mild conditions (low critical pressure and temperature), extraction occurs in a closed system without oxygen^22^. Because of the inert atmosphere, the absence of light, and the short extraction time, this method provides significant benefits for the extraction of pigments from *R. tinctorum* plants. Furthermore, SFE is more selective than conventional extraction methods and strives to enhance the recovery and quality of the extracted components. CO_2_ is commonly used as a solvent in SFE. CO_2_ is a low-cost, non-flammable, and non-toxic readily available solvent that, owing to its high volatility, can be easily removed from the final extract while maintaining the biological qualities of bioactive substances as an alternative to other solvents^7,23–26^. The co-solvents may enhance CO_2_ solubilizing capacity and improve polar molecule solubility^27^. A considerable amount of research is being conducted worldwide on extraction under scCO_2_^28–33^. As a result of the formation of complexes between the functional groups of the dye and fabrics, chemical mordants such as metallic salts are commonly used in textile dyeing to improve shade depth and fastness^34^. Heavy metal ions generated during the dyeing process may pose health and environmental hazards^35,36^. As a result, tannin-rich plants have recently become popular as bio-mordants^34,37–39^.

This study aimed to extract alizarin pigment from *R. tinctorum* using SFE technology as a green alternative to conventional extraction methods. Furthermore, various parameters, including the co-solvent ratio, temperature, pressure, extraction time, and flow rate, were optimized to maximize alizarin recovery and content. Wool fabrics were then dyed using *R. tinctorum* root extract obtained under optimal extraction conditions using the conventional dyeing process. In this study, the effects of extract concentration and chemical and natural mordants on the coloring properties of dyed wool fabrics were evaluated. Furthermore, RE and dyed wool fabrics were evaluated for their antibacterial properties.

## Experimental sections

### Materials

*R. tinctorum* roots were cultivated and harvested from the Faculty of Agriculture, Damietta University, Egypt. The collection of plant roots was complied with relevant institutional, national, and international guidelines and legislation. To remove impurities, they were thoroughly washed with distilled water and dried at room temperature. Using a laboratory-scale mill (M 20; IKA, Staufen, Germany), the dried roots were ground. Bleached wool fabrics (100%) were provided by Misr for the spinning and weaving Company, Mahalla El-Kobra, Egypt. The solvent used for the supercritical fluid extraction was CO_2_ (99.6%, supplied by NETCO Industrial Company, Cairo, Egypt), and the co-solvent was gradient-grade methanol (99.8% purity). For high-performance liquid chromatography analysis, alizarin red, HPLC-grade acetonitrile (99.8%) and ammonium acetate were used. As part of the conventional dyeing process, aluminum sulphate was used as a chemical mordant, anionic dispersing agent (Dispex, DyStar, Cairo, Egypt), and acetic acid. Chemicals were purchased from Sigma-Aldrich for this study.

### Procedures

#### Extraction of alizarin pigment using scCO_2_

The supercritical CO_2_ extraction system consisted of a CO_2_ cylinder, a chiller (model Julabo FL601), a semi-preparative CO_2_ pump (model JASCO PU-4386), a co-solvent pump (model JASCO PU-4180), an interface box (model LC-NeII/ADC), a pressure regulator (model JASCO BP-4340), a heater controller (model HC-2068–01), a temperature and speed controller (model EYELARCX-1000 H), a 50 mL high-pressure stainless steel vessel, and a vial for extract collection (model HE-4340-01-200). The maximum flow rate of the circulation pump was 10 mL/min^40^.

In the extraction process, approximately 4 g of dried *R. tinctorum* roots was placed in the vessel. Subsequently, the system was closed. The chiller was set to − 5 °C, and CO_2_ was cooled to maintain liquid conditions. All the extraction parameters (composition ratio, temperature, pressure, time, and flow rate) were controlled using a software system. Liquid CO_2_ was preheated using a heating jacket to reach set temperature levels. The extraction procedure consists of two phases: static and dynamic. The static time used in this study was approximately 20 min. During static time, scCO_2_ was supplied to the extractor, while the outlet back pressure valve was closed. Static mode is the time at which the plant is exposed to a constant scCO_2_ amount for a certain time. This allows scCO_2_ to penetrate the plant matrix and dissolve target components. After the static time, the valve was adjusted to allow cooled, liquefied scCO_2_ to pass through the extracting vessel (dynamic conditions). In the dynamic mode, the plant matrix is regularly fed with fresh CO_2_, and the extracted components are moved from the extraction vessel into the collecting system^41,42^.

After the extraction time had expired, the temperature was reduced to room temperature and the pressure was reduced to atmospheric level. This decomposition causes scCO_2_ to lose its solubility and return to the gas phase. All extracts were collected in collection vials and stored in a freezer at − 20 °C until further analysis.

#### Determination of alizarin using high-performance liquid chromatography (HPLC)

Chromatographic measurements were performed using a high-performance liquid chromatography system (JASCO, Tokyo, Japan). This system was equipped with an SFC autosampler (model AS-4350), RHPLC pump (model PU-4180), CO_2_ pump (model PU-4380), interface box (model LC-NeII/ADC), UV/Vis detector (model UV-4070), column oven (model CO-4060), back-pressure regulator (model BP-4340), microsorb column-MV 100-5 C18 250 × 4.6 mm (Agilent Technologies, Santa Clara, USA), and JASCO software for determination and data analysis.

For the quantitative determination of alizarin, five standard solutions of alizarin at different concentrations (25, 50, 100, 150, and 200 μg/mL) were prepared and dissolved in a mixture of methanol and distilled water at a volume ratio of 50:50. Calibration graphs were constructed by plotting the peak areas of alizarin.

The mobile phase consisted of a mixture of acetonitrile and ammonium acetate (20 mM) at a ratio of 60:40 (v/v). Before use, the mobile phase was prepared, filtered using a micro syringe filter (0.45μm), and degassed using an ultrasonicator (model S30H, Elmasonic, Singen, Germany) for 20 min. The injection volume was 20 μL in triplicate. The elution program was performed for 15 min at 30°C. Detection was performed at an absorbance of 254 nm. The flow rate was set at 1 mL/min^43^.

#### Preparation of bio-mordants

The peels of *P. granatum*, flowers of *Rhus coriaria* (*R. coriaria*), and powdered leaves of *Camellia sinensis* (*C. sinensis*) were utilized as biomordants during wool dyeing. They belong to the families *Lythraceae*, *Anacardiaceae*, and *Theaceae*, respectively. The peels of the *P. granatum* and *R. coriaria* flowers were washed with distilled water and dried at room temperature. Dried peels and flowers were ground into a fine powder. *P. granatum* peel, *R. coriaria* flowers, and *C. sinensis* leaves were purchased from a local market in Damietta, Egypt.

#### Dyeing process

The wool fabric was scoured in a water bath containing 2 g/L non-ionic detergent (Sera Fast CRD, DyStar, Cairo, Egypt) for 15 minutes, rinsed, and dried at room temperature before mordanting. At a liquor-to-fabric ratio of 1:40, the fabric was pre-mordanted in a bath containing 50% weight of fabric (OWF) bio-mordant or 5% (OWF) aluminum sulfate as a chemical mordant. Over a period of 20 minutes, scouring wool was added to the mordanting bath at 25 °C and the temperature was gradually increased to 45 °C. At this temperature, mordanting was performed for 30 minutes. Subsequently, the samples were thoroughly rinsed and dried. The dye, RE, was dispersed by adding 2 g/L of an anionic dispersing agent. Acetic acid was used to adjust the pH to 3. Various RE concentrations of 2, 4, and 6% (OWF) were used to dye mordanted wool. Over 30 minutes, the temperature was increased from 45 °C to 90 °C, and then maintained at 90 °C for 60 minutes. The fabric was washed with cold water after dyeing. The sample was soaped with a non-ionic detergent at a concentration of 2 g/L at 70 °C for 20 min, rinsed, and dried at room temperature.

#### Evaluation of color characteristics and fastness properties

A spectrophotometer (Konica Minolta, CM-3600, Tokyo, Japan) was used to measure color strength (*K/S* values) and color coordinates in terms of CIELab (lightness [L*], redness-greenness value [a*], yellowness-blueness value [b*], chroma [C*], and hue [h]). After calibrating the instrument, each sample was assayed at three positions (left, central, and right). The color strength (*K/S*) values were determined in the visible region of the spectrum (400–700 nm) according to the Kubelka –Munk equation, as follows:

1 $$ K/S = \left( {1 \, - \, R} \right)^{2} /\left( {2R} \right) $$

This equation describes the relationship between reflectance and absorbance, where R is the reflectance of the dyed sample, K is the absorption coefficient and S is the scattering coefficient.

The color difference between any dyed and blank sample was calculated as the square root of the squares of the corresponding L*, a*, and b* differences, as follows:

2 $$ \Delta E* = \sqrt {\left( {L_{2}^{*} - L_{1}^{*} } \right)^{2} + \left( {a_{2}^{*} - a_{1}^{*} } \right)^{2} + \left( {b_{2}^{*} - b_{1}^{*} } \right)^{2} } $$

where ∆E is the difference between the blank wool and dyed wool fabrics, a* is the red (+)/green (-) ratio, b* is the yellow (+)/blue (-) ratio, and L* is the lightness from black (0) to white (100). The leveling properties of the dyed wool fabrics were investigated. The UV/Vis spectrum of alizarin in the dye bath was measured before and after the dyeing process using a UV/Vis spectrophotometer (Alpha-1860, Noble, IN, USA). The color fastness of the dyed wool samples was assessed according to ISO standard methods. The specific tests were as follows: (AATCC Test Method 61-1996) for wash fastness^44^, (AATCC Test Method 61 (2A)-1996) for durability testing^45^, (AATCC Test Method 16-2004) for light fastness^46^, and (AATCC Test Method 8-2001) for dry and wet rubbing fastness^47^. Color changes and staining were assessed using grayscale scores ranging from 1 (worst) to 5 (best).

#### Antibacterial activity

Wool fabrics dyed with RE and RE obtained via the SFE technique were evaluated according to AATCC (147-2004)^48^. Gram-positive (*Bacillus cereus*) and gram-negative (*E. coli*, *Salmonella typhi*, and *P. aeruginosa*) bacteria were tested for antibacterial activity. Dimethyl sulfoxide (DMSO) was used as a solvent to dissolve the extract at a concentration of 10 mg/mL to evaluate its antibacterial activity. The dyed samples were cut into squares with dimensions of 10 mm × 10 mm. To compare the antibacterial activities of standard antibiotics with those of the tested bacteria, tetracycline (30 g) and ciprofloxacin (10 g) were also tested. At 37°C, the bacterial strains were incubated for 24 hours. A growth inhibition zone diameter (mm) was determined after incubation^49,50^.

### Statistical analysis

A Costat program version 6.311 (CoHort software, Monterey, USA) was used to analyze the data obtained from this study. Statistical analysis of variance (ANOVA) was used to compare all samples. The standard deviation (SD) was calculated, and significant variations among all means were analyzed using Duncan’s new range test at *P* = 0.05^51–53^. Furthermore, each sample was statistically analyzed three times.

## Results and discussion

### Design of experiment

Several experiments were conducted to study the effect of different parameters on alizarin recovery and its content in RE. Among these parameters were a solvent composition ratio of CO_2_: Me (50%:50%, 70%:30%, 80%:20%, 90%:10%, and 100%:0%), temperature (45, 55, 65, 75, 85, and 95°C), pressure (150, 175, 200, 225, and 250 bar), extraction time (15, 30, 45, 60, 90, and 120 min), and flow rate (5,7, and 9 mL/min). Based on alizarin recovery and minimizing environmental risk, the optimum conditions for alizarin extraction were selected. HPLC UV/Vis was used to analyze the alizarin concentration in RE.

### Evaluation of the optimum conditions for alizarin extraction

**Figure 1:**
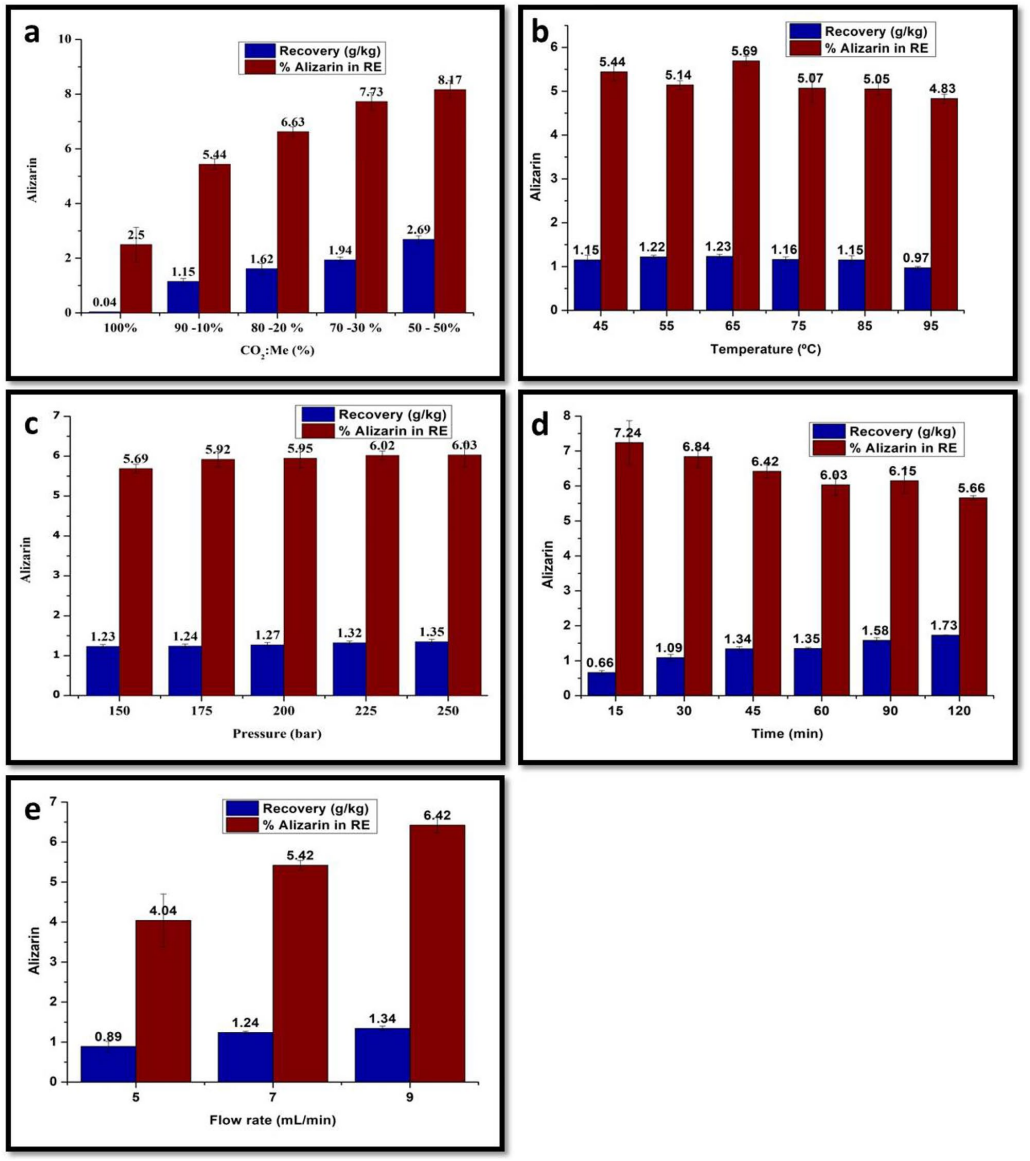
Effects of (**a**) solvent composition ratio, (**b**) temperature, (**c**) pressure, (**d**) extraction time, (**e**) flow rate on alizarin recovery (g/kg roots), and alizarin content (%) in RE.

We investigated the effect of various parameters on the efficiency of alizarin extraction from *R. tinctorum* roots using scCO_2_. The optimization of extraction conditions is a crucial step in the development of the SFE method. Thus, these parameters, such as temperature, pressure, time, and flow rate, were studied and analyzed statistically, as shown in Table 1 and Fig. 1.

**Table 1:** The alizarin yield and statistical analysis of different SFE conditions (^a–m^Means within a column followed by the same letter(s) are not significantly different according to Duncan’s multiple range test (*P* = 0.05)).

Experiment conditions						Alizarin (g per Kg roots)	Alizarin% (g per100 g extract)
Composition ratio		Temperature (ºC)	Pressure (bar)	Time (min)	Flow rate (mL/min)		
CO_2_ (%)	Me (%)						
100	–	45 ± 1	150 ± 0.5	60	9	0.04 ± 0.00 i	2.50 ± 0.63 m
90	10					1.15 ± 0.11 f.	5.44 ± 0.20 hij
80	20					1.62 ± 0.19 cd	6.63 ± 0.18 de
70	30					1.94 ± 0.10 b	7.73 ± 0.32 ab
50	50					2.69 ± 0.13 a	8.17 ± 0.33 a
90	10	45 ± 1	150 ± 0.5	60	9	1.15 ± 0.11 f.	5.44 ± 0.20 hij
		55 ± 1				1.22 ± 0.04 ef	5.14 ± 0.10 ijk
		65 ± 1				1.23 ± 0.05 ef	5.69 ± 0.11 ghi
		75 ± 1				1.16 ± 0.06 f.	5.07 ± 0.33 jk
		85 ± 1				1.15 ± 0.10 f.	5.05 ± 0.14 jk
		95 ± 1				0.97 ± 0.03 g	4.83 ± 0.10 k
90	10	65 ± 1	150 ± 0.5	60	9	1.23 ± 0.05 ef	5.69 ± 0.11 ghi
			175 ± 0.5			1.24 ± 0.05 ef	5.92 ± 0.20 fgh
			200 ± 0.5			1.27 ± 0.06 ef	5.95 ± 0.24 fgh
			225 ± 0.5			1.32 ± 0.05 e	6.02 ± 0.11 fg
			250 ± 0.5			1.35 ± 0.03 e	6.03 ± 0.30 fg
90	10	65 ± 1	250 ± 0.5	15	9	0.66 ± 0.06 h	7.24 ± 0.63 bc
				30		1.09 ± 0.09 f.	6.84 ± 0.31 cd
				45		1.34 ± 0.06 e	6.42 ± 0.19 def
				60		1.35 ± 0.03 e	6.03 ± 0.30 fg
				90		1.58 ± 0.08 d	6.15 ± 0.35 efg
				120		1.73 ± 0.01 c	5.66 ± 0.06 ghi
90	10	65 ± 1	250 ± 0.5	45	5	0.89 ± 0.13 g	4.04 ± 0.66 l
					7	1.24 ± 0.03 ef	5.42 ± 0.12 hij
					9	1.34 ± 0.06 e	6.42 ± 0.19 def

#### Effect of solvent composition ratio (CO_2_: Me)

The co-solvent in this study was methanol because it is the most selective solvent for the extraction of alizarin^54^. The influence of the co-solvent ratio on the extraction yield of alizarin was the most significant, as shown in Table 1. Alizarin recovery and its percentage in the RE increased as the co-solvent ratio increased. Alizarin contains hydroxyl groups that form hydrogen bonds with other methanol hydroxyl groups, resulting in this effect. Solvent polarity and density are increased by methanol. Moreover, the co-solvent causes matrix swelling, which facilitates solvent–compound contact and acts as a carrier for alizarin^55^. As a result of its toxic effects, an increase in the methanol ratio poses an environmental risk. Using a solvent composition ratio of 90% CO_2_:10% Me led to a significant increase in alizarin recovery (1.15 g/kg) over extraction without co-solvent addition (0.04 g/kg). The extract obtained at a ratio of 90% CO_2_:10% Me contained more alizarin in RE (5.44%) than that obtained at a ratio of 100% CO_2_. As the methanol ratio increased, both alizarin recovery and its content in RE increased. On the other hand, the amount of toxic organic solvent (methanol) used was reduced to 10% instead of 50% from an environmental perspective. In order to avoid the harmful effects of large amounts of organic solvents, optimal conditions of temperature, pressure, extraction time, and flow rate were investigated in order to maximize alizarin recovery at a low co-solvent ratio (10% Me).

#### Effect of temperature on alizarin extraction efficiency

Extraction efficiency is heavily influenced by temperature. In this study, alizarin recovery was determined at different temperatures between 45 and 95 °C under constant conditions for all other parameters. As the temperature increased above 65 °C, the recovery of alizarin decreased, and as the temperature decreased, the recovery increased. The temperature affects extraction in two ways: it reduces solvent viscosity and increases diffusivity of the compound, thereby improving extraction efficiency. The density of scCO_2_ can be reduced at high temperatures as a result of thermal degradation of matrix components or excessive co-extraction^55^. In order to minimize the degradation of thermolabile compounds, the supercritical fluid extraction temperature should be adjusted between 35 and 60 °C. As shown in Fig. 1, 65 °C was the most suitable temperature for alizarin extraction from *R. tinctorum* roots. For further optimization of pressure, extraction time, and flow rate parameters, the optimum temperature (65 °C) was adjusted.

#### Effect of pressure on the efficiency of alizarin extraction

The pressure is another parameter that affects the extraction efficiency of alizarin using the SFE technique. To study the effect of pressure on the extraction efficiency of alizarin, dried *R. tinctorum* roots were extracted under various pressure levels (150, 175, 200, 225, and 250 bar). The co-solvent ratio, temperature, extraction time, and flow rate were all constant at 90 CO_2_:10 Me, 65°C, 60 minutes, and 9 mL/min, respectively.

As pressure increased, alizarin recovery increased. Under 150 bar pressure, alizarin recovery from *R. tinctorum* roots was approximately 1.23 g/kg roots and increased to 1.34 g/kg under 250 bar pressure. In addition, the alizarin content in the RE With an increase in pressure from 150 bar to 250 bar, the alizarin content in the RE increased from 5.69 to 6.18% (Fig. 1 and Table 1). The density and diffusivity of CO_2_ increased with increasing pressure. By increasing pressure, the fluid interacts with the matrix, which improves the solubility and extraction efficiency of alizarin. Based on the optimization study of other extraction parameters, including time and flow rate, the optimum pressure level of 250 bar was selected to maximize alizarin extraction efficiency*.*

#### Effect of extraction time on the efficiency of alizarin extraction

The effect of extraction time on alizarin extraction efficiency can be used to determine the optimum time for alizarin recovery in SFE. An incomplete extraction can occur if the extraction time is too short. Conversely, if the extraction time is too long, it wastes time and solvent, resulting in a higher extraction cost^56,57^. At a constant flow rate of 9 mL/min, *R. tinctorum* roots were extracted for 15, 30, 45, 60, 90, and 120 min, with all other parameters set to their optimized conditions. As extraction time increased, alizarin recovery increased from 0.66 to 1.73 g/kg roots.

After 45 min, the rate of increase slowed down. To save time and money, we conducted the following study on flow rate optimization for 45 minutes^58,59^. The increased contact time between scCO_2_ and the sample improved extraction efficiency and maximized alizarin content in the RE. As a result of the extraction of other components from *R. tinctorum* roots besides alizarin, the percentage value of alizarin in the RE decreased with time (Fig. 1and Table 1).

#### Effect of flow rate on the efficiency of alizarin extraction

As a final step, the flow rate of the supercritical fluid was investigated in relation to the alizarin extraction efficiency, either the recovery or the content of alizarin in the RE obtained using the SFE technique. An optimization study of the flow rate determined the optimal composition ratio, temperature, pressure, and extraction time of 90% CO_2_:10% Me, 65°C, 250 bar, and 45 min. For alizarin extraction using SFE, flow rates of 5, 7, and 9 mL/min were tested. Increasing flow rate of supercritical fluid resulted in a significant increase in alizarin recovery from 0.89 to 1.34 g/kg roots. Additionally, the alizarin content in the extract increased with increasing flow rate, as shown in Fig. 1 and Table 1. By increasing the flow rate, the mass transfer resistance around solid particles decreases, allowing the solute to be easily transported to the supercritical fluid, leaving the extractor vessel saturated. As a result, alizarin extraction becomes more efficient^56^. According to the results, co-solvent ratio, flow rate, and extraction time were more effective for alizarin recovery and content than temperature and pressure. In the root samples extracted at various pressure levels, there were no significant differences in pressure, as shown in Fig. 1 and Table 1.

### Testing of dyed wool fabrics

#### Evaluation of color and fastness properties

**Figure 2:**
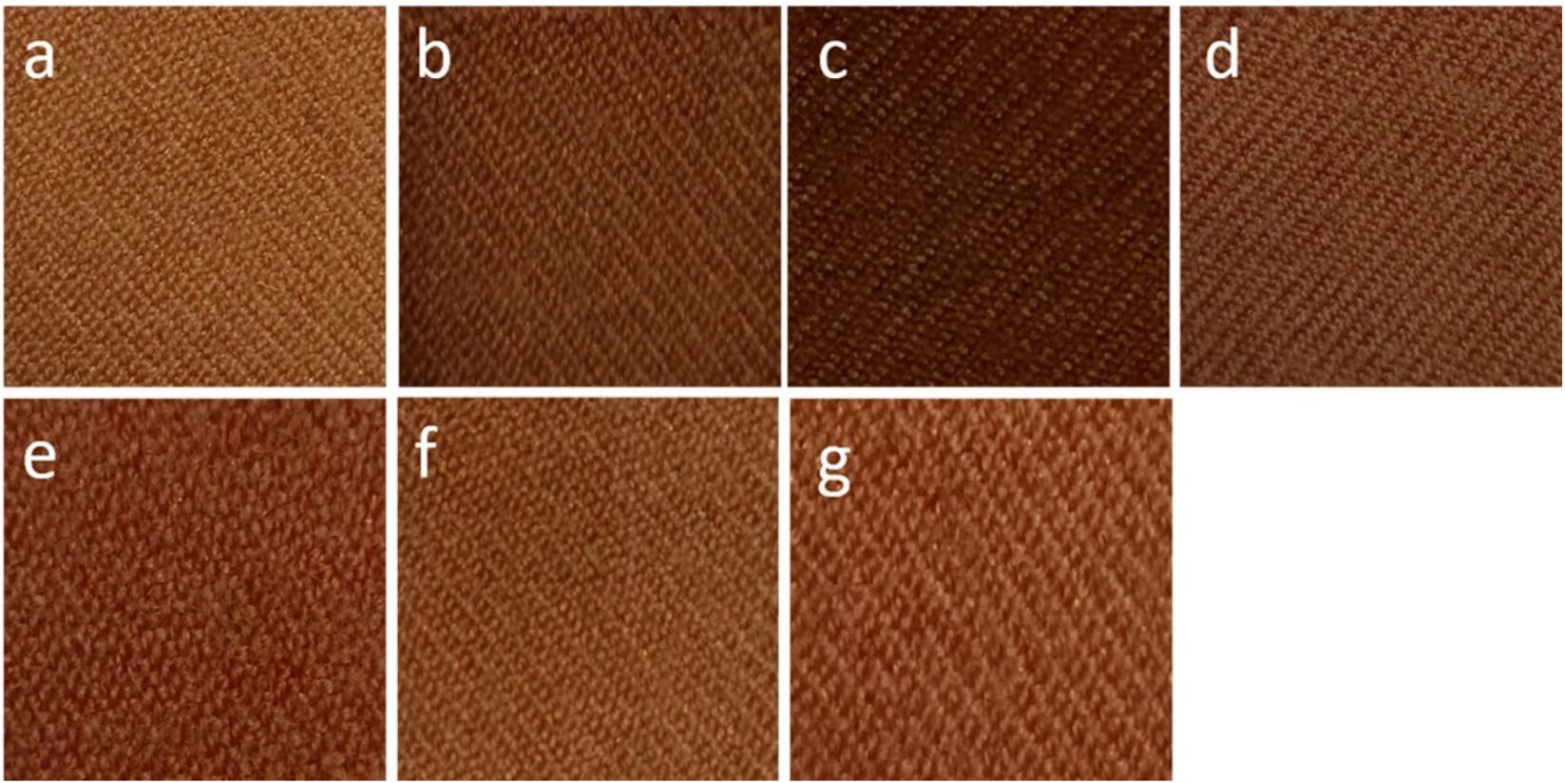
Wool fabrics dyed using (**a**) 2% RE without mordant, (**b**) 4% RE without mordant, (**c**) 6% RE without mordant, (**d**) 4% RE with Al_2_(SO_4_)_3_, (**e**) 4% RE with *P. granatum*, (**f**) 4% RE with *C. sinensis*, (**g**) 4% RE with *R. coriaria*.

The colorimetric properties of wool dyed with RE are listed in Table 2. Alizarin (the main pigment in RE) produced rich colors on wool fabrics. Based on the results in Table 2, all dyed samples were found to be in the red-yellow zone (a > b). Depending on the RE concentration and the mordant used, the samples take on an orange hue. After adding the mordant, the shades were reddish-brown for Al_2_(SO_4_)_3_, heavily reddish-orange for *P. granatum*, brown for *C. sinensis*, and bright orange for *R. coriaria*, as shown in Fig. 2. By comparing the mordanted and unmordanted samples, it was found that the color strength (*K/S*) values increased, and the lightness (L*) and chroma (C*) values increased in brighter shades. This increase in *K/S* can be attributed to the fact that *P. granatum* peel, *R. coriaria*, and *C. sinensis* are rich in tannins. The phenolic hydroxyl groups of tannins form hydrogen bonds with the reactive groups in wool fabrics. These bonds improve the adsorption of alizarin on the fabric^14^. The color difference (ΔE) was calculated, which revealed excellent leveling characteristics.

**Table 2:** Colorimetric properties of wool samples dyed with RE (Color lightness (L*), the degree of redness (+ ve) and greenness (-ve) (a*), and the degree of yellowness (+ ve) and blueness (-ve) (b*), the color strength (*K/S*)).

Type	Color parameters							
Mordant	RE concentration (%)	L*	a*	b*	∆E*	C*	h	*K/S* at 360 nm
–	2	30.06 ± 0.32 c	5.58 ± 0.16 e	5.87 ± 0.18 d	56.83 ± 0.32 c	8.1 ± 0.19 d	46.48 ± 0.96 a	9.97 ± 0.14 d
–	4	31.50 ± 0.36 b	8.58 ± 0.39 d	7.64 ± 0.21 c	55.73 ± 0.29 d	11.49 ± 0.43 c	41.68 ± 0.57 bc	10.46 ± 0.11 c
–	6	26.70 ± 0.48 d	4.12 ± 0.46 f.	4.17 ± 0.48 e	60.23 ± 0.49 a	5.86 ± 0.67 e	45.36 ± 0.04 a	11.13 ± 0.16 b
Al_2_(So_4_)_3_	4	32.47 ± 0.35 a	9.89 ± 0.07 c	8.90 ± 0.38 b	54.97 ± 0.29 e	13.30 ± 0.26 b	41.97 ± 1.27 b	10 ± 0.11 d
*P. granatum*	4	30.44 ± 0.52 c	12.15 ± 0.31 a	10.18 ± 0.26 a	57.40 ± 0.44 b	15.85 ± 0.39 a	39.96 ± 0.21 d	12.11 ± 0.25 a
*C. sinensis*	4	32.24 ± 0.11 a	12.24 ± 0.21 a	10.42 ± 0.20 a	55.67 ± 0.15 d	16.07 ± 0.28 a	40.40 ± 0.16 cd	11.06 ± 0.12 b
*R. coriaria*	4	29.89 ± 0.11 c	11.35 ± 0.40 b	7.77 ± 0.60 c	57.83 ± 0.06 b	13.75 ± 0.66 b	34.34 ± 1.19 e	11.27 ± 0.18 b

a–fMeans within a column followed by the same letter(s) are not significantly different according to Duncan’s multiple range test (*P* = 0.05).

**Figure 3:**
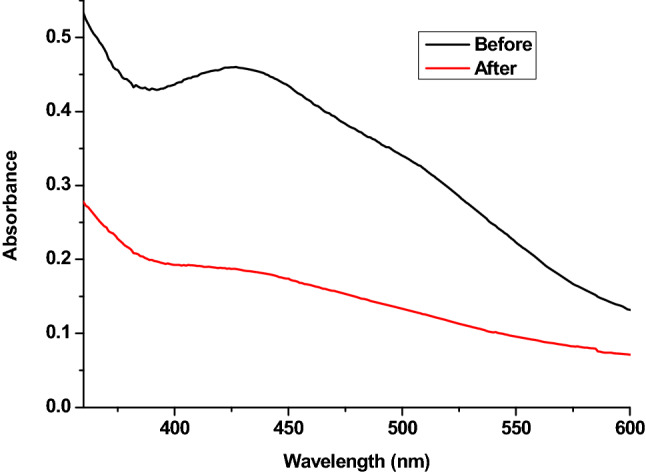
UV/ Visible spectrum of RE dyeing solution before and after dyeing.

Figure 3 depicts the absorbance of alizarin before and after exhaustion by the wool fabric in the range of 200–800 nm. The spectrum of alizarin before dyeing showed a sharp absorption band at 445 nm^60,61^, indicating the presence of the alizarin pigment. After dyeing, the spectrum of alizarin clearly decreased, confirming the exhaustion of alizarin by wool fabric.

The color fastness, including washing, light, and rubbing, was evaluated according to grayscale, and is presented in Table 3. Even after the durability test, dyed samples (without mordant and with mordant) showed excellent wash fastness (4-5 or 5). Fastness to dry and wet rubbing was good to excellent (4–5). The fastness to light ranged from good to very good.

**Table 3:** Fastness properties of wool samples dyed with RE (Wash, light and rubbing fastness on gray scale: 1, poor; 2, fair; 3, moderate; 4, good; 5, excellent).

Sample		Fastness properties of dyed wool						
Mordant	RE concentration (%)	Wash Fastness		Durability (Up to 5 washing cycles)		Rubbing fastness		Light fastness
		Color change	Staining	Color change	Staining	Wet	Dry	
**–**	2	4–5	5	4	4–5	4	4–5	4–5
**–**	4	4–5	5	4	4–5	4	4	4–5
**–**	6	4–5	5	4	4–5	4	4	4–5
Al_2_(So_4_)_3_	4	4–5	5	4–5	4–5	4–5	4–5	4
*P. granatum*	4	4–5	5	4	4–5	4–5	5	4
*C. sinensis*	4	4–5	5	4–5	4–5	5	5	4–5
*R. coriaria*	4	4–5	5	4–5	4–5	4–5	4–5	4

#### Antibacterial activity

RE (10 mg/mL) and wool fabrics dyed with 4% RE and *P. granatum* peels were tested against gram-positive (*B. cereus*) and gram-negative (*E. coli, S. typhi, and P. aeruginosa*) bacteria. Under optimum conditions, the SFE extract was highly effective against *P. aeruginosa* and *E. coli* with inhibition zones of 23 and 15 mm, respectively. As shown in Table 4, it showed low antibacterial activity against *B. cereus* and *S. typhi*. Furthermore, *P. aeruginosa* was more sensitive to wool dyed with RE than other bacteria tested. The lowest antibacterial activity was observed against *B. cereus*, with a 3 mm diameter inhibition zone. A blank wool fabric showed no antibacterial activity, confirming that RE mediated the antibacterial activity of dyed fabrics. The antibacterial effect is attributed to the release of alizarin molecules, which affect the bacterial cell wall and inhibit bacterial growth^13,62^.

**Table 4:** Diameters of inhibition zones (mm) of the extract and dyed wool fabrics at 37 °C for 24 h compared with standard compounds.

Sample	*B. cereus* (G+)	*E. coli* (G−)	*P. aeruginosa* (G−)	*S. typhi* (G−)
Extract (10 mg/mL)	7 ± 0.6 c	15 ± 0.6 d	23 ± 1.5 a	8 ± 0.6 c
Wool fabric (blank)	0 ± 0.0 e	0 ± 0.0 e	0 ± 0.0 d	0 ± 0.0 d
Dyed wool fabric	3 ± 0.6 d	17 ± 0.6 c	20 ± 1.5 b	13 ± 1.0 b
Ciprofloxacin (10 μg)	19 ± 1.0 a	22 ± 1.0 a	17 ± 1.5 bc	15 ± 1.5 a
Tetracycline (30 μg)	15 ± 1.0 b	19 ± 2.0 b	16 ± 2.0 c	13 ± 0.6 b

a–eMeans within a column followed by the same letter(s) are not significantly different according to Duncan’s multiple range test (*P* = 0.05).

## Conclusions

By using scCO_2_, SFE was demonstrated to be an eco-friendly method for obtaining alizarin from *R. tinctorum* roots. By examining the effects of co-solvent ratio, temperature, pressure, extraction time, and flow rate, the optimal extraction conditions for alizarin were determined. The co-solvent ratio was the most important factor influencing alizarin extraction yield. Based on alizarin recovery and minimizing environmental impact, SFE conditions of 90% CO_2_:10% Me, 65°C, 250 bar, 45 min, and 9 mL/min were selected. The wool fabrics were dyed with RE obtained under optimal conditions at different concentrations (2–6%). Dyed fabrics with bio-mordants had better color and fastness properties than those with Al_2_(SO_4_)_3_. Also, dyed fabrics and RE were tested for their antibacterial properties. In comparison with other bacterial strains, dyed fabric and RE were more effective against *P. aeruginosa*. With SFE, it is possible to extract natural colorants with supercritical technology, minimizing the environmental impacts of organic solvents.

## Supplementary Information

Supplementary Information.

## Data Availability

All data generated or analyzed during this study are included in this published article [and its supplementary information files].
