# Supplementary material for: An eco-friendly method of extracting alizarin from Rubia tinctorum roots under supercritical carbon dioxide and its application to wool dyeing
Source: Sci Rep. 2023 Jan 2;13:30. doi: 10.1038/s41598-022-27110-0 (PMC9807584; doi:10.1038/s41598-022-27110-0)
Supplement: Supplementary file 1 — Supplementary Information. [file 41598_2022_27110_MOESM1_ESM.pdf]

Standard curve of alizarin

Alizarin	RT	Area (μV.sec)	conc. (μg/ml)
	2.11	220242	25
	2.21	528150	50
	2.08	1060998	100
	2.06	1676062	150
	2.1	2170979	200

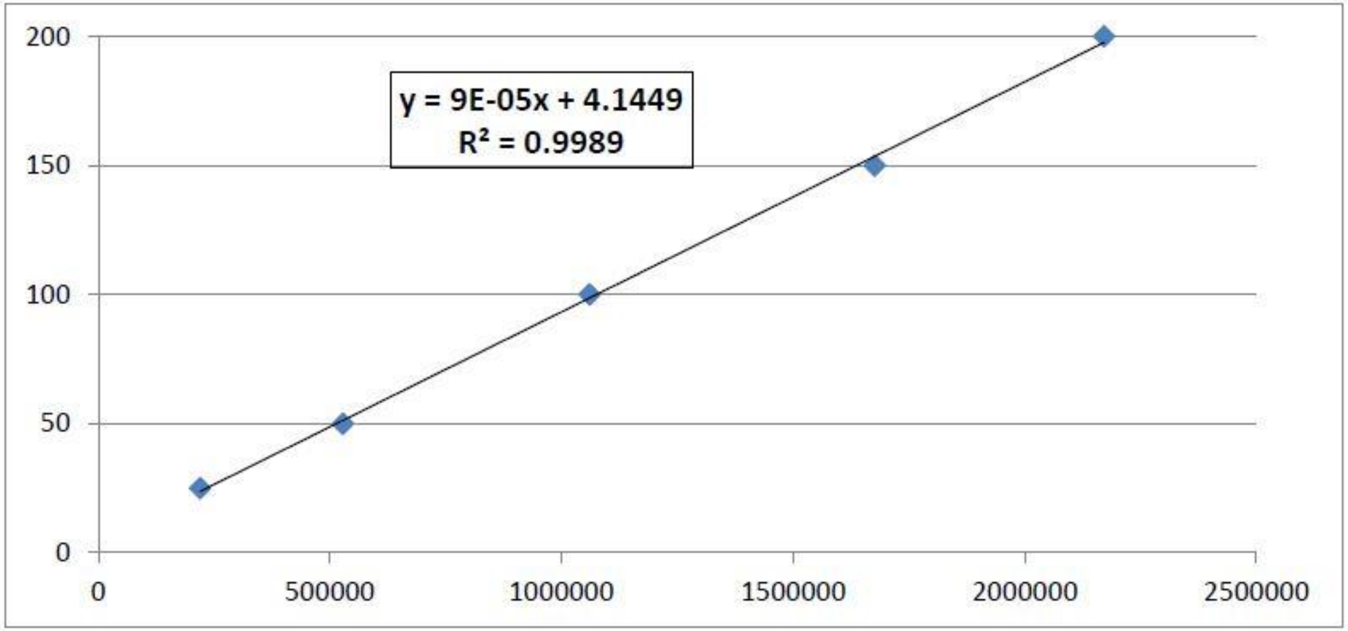

Color measurement

No.	Mordant	RE	L*(D65)	a*(D65)	b*(D65)	K/S Val(360)	K/S Val(370)	K/S Val(380)	K/S Val(390)	K/S Val(400)	K/S Val(410)	K/S Val(420)	K/S Val(430)	K/S Val(440)	K/S Val(450)
1-1	-	2%	30.18	5.4	5.85	9.89257	9.77594	9.63926	9.57222	9.50603	9.41906	9.2703	9.22858	9.14616	9.12576
1-2			29.69	5.63	5.71	10.13361	10.08455	9.98771	9.9162	9.79906	9.68443	9.55007	9.55007	9.44067	9.35478
1-3			30.3	5.7	6.06	9.86904	9.79906	9.6618	9.55007	9.48415	9.37612	9.31237	9.2494	9.1872	9.12576
2-1	-	4%	31.3	8.42	7.5	10.516	10.18311	9.84561	9.63926	9.55007	9.44067	9.33353	9.2494	9.14616	8.98526
2-2			31.92	9.03	7.88	10.33432	9.96377	9.6618	9.46236	9.31237	9.1872	9.12576	8.96551	8.79123	8.71572
2-3			31.29	8.3	7.54	10.516	10.18311	9.9162	9.72999	9.55007	9.46236	9.37612	9.2703	9.10545	8.98526
3-1	-	6%	26.15	3.59	3.63	11.01126	11.06894	11.186	11.2753	11.24539	11.21562	11.24539	11.2753	11.30536	11.30536
3-2			26.93	4.29	4.35	10.81365	10.86944	10.92576	10.98262	10.95412	11.01126	11.01126	11.01126	11.01126	10.98262
3-3			27.03	4.47	4.53	10.92576	10.89753	10.86944	10.92576	10.95412	11.01126	11.06894	11.04003	11.06894	10.95412
4-1	$Al_2(So_4)_3$	4%	32.69	9.97	8.96	9.89257	9.68443	9.46236	9.31237	9.16664	9.04499	8.94583	8.86792	8.75333	8.60464
4-2			32.07	9.86	8.49	10.01176	9.82229	9.59447	9.39754	9.2703	9.1872	9.08521	9.06506	8.94583	8.84863
4-3			32.66	9.83	9.25	10.10902	9.86904	9.6618	9.50603	9.35478	9.2703	9.14616	8.98526	8.86792	8.73449
5-1	P. granatum	4%	29.84	11.85	9.89	12.38768	12.31667	12.14241	11.93925	11.7747	11.71006	11.55128	11.36593	11.30536	11.2753
5-2			30.8	12.46	10.39	11.906	11.7423	11.61431	11.45791	11.24539	11.09799	10.92576	10.86944	10.75839	10.64941
5-3			30.68	12.14	10.26	12.04003	12.00626	11.71006	11.48888	11.4271	11.186	11.06894	10.95412	10.81365	10.64941
6-1	C. sinensis	4%	32.24	12.18	10.29	10.95412	10.81365	10.64941	10.4635	10.23306	10.03592	9.9162	9.77594	9.55007	9.48415
6-2			32.14	12.47	10.65	11.186	11.01126	10.81365	10.62248	10.38564	10.20803	10.08455	9.9162	9.82229	9.70716
6-3			32.35	12.07	10.31	11.04003	10.84148	10.56899	10.35992	10.13361	9.96377	9.84561	9.6618	9.55007	9.35478
7-1	R. coriaria	4%	29.94	11.53	8.17	11.39644	11.15652	10.89753	10.75839	10.67646	10.56899	10.43743	10.38564	10.41147	10.25821
7-2			29.96	11.63	8.05	11.33557	11.04003	10.84148	10.75839	10.64941	10.516	10.35992	10.35992	10.23306	10.20803
7-3			29.76	10.9	7.08	11.06894	10.73095	10.48969	10.35992	10.25821	10.18311	10.10902	10.13361	9.9162	9.86904
8-1	Blank		86	-0.86	11.15	1.10792	0.79969	0.61078	0.49466	0.40968	0.33908	0.27983	0.23525	0.19886	0.17373
8-2			86.67	-0.86	11.09	1.00096	0.71988	0.55269	0.45158	0.37537	0.31196	0.25899	0.21766	0.1834	0.16116
8-3			86.38	-0.7	11.27	1.0705	0.77785	0.59893	0.48549	0.40024	0.32948	0.27148	0.22768	0.19243	0.16842

No.	Mordant	RE	K/S Val(460)	K/S Val(470)	K/S Val(480)	K/S Val(490)	K/S Val(500)	K/S Val(510)	K/S Val(520)	K/S Val(530)	K/S Val(540)	K/S Val(550)	K/S Val(560)	K/S Val(570)
1-1	-	2%	8.92624	8.77224	8.60464	8.4251	8.46046	8.40751	8.11841	7.8158	7.64969	7.51841	7.21386	6.73661
1-2			9.1872	9.12576	8.94583	8.77224	8.86792	8.75333	8.47825	8.10196	7.95657	7.83122	7.51841	6.98058
1-3			8.92624	8.77224	8.60464	8.4251	8.46046	8.39	8.15149	7.78512	7.60548	7.50408	7.18726	6.67796
2-1	-	4%	8.75333	8.532	8.40751	8.30339	8.30339	8.26919	8.13492	7.7244	7.46135	7.29471	6.93054	6.26003
2-2			8.47825	8.30339	8.15149	8.03676	7.98847	8.00451	7.89345	7.48979	7.22722	7.06969	6.67796	5.99005
2-3			8.79123	8.62298	8.40751	8.28626	8.26919	8.25219	8.11841	7.70936	7.47555	7.29471	6.89344	6.24966
3-1	-	6%	11.15652	11.04003	10.98262	10.84148	10.73095	10.70364	10.54244	10.33432	10.08455	9.89257	9.57222	9.1872
3-2			10.84148	10.64941	10.62248	10.43743	10.35992	10.30883	10.10902	9.86904	9.63926	9.39754	9.08521	8.64138
3-3			10.84148	10.62248	10.59567	10.38564	10.35992	10.33432	10.10902	9.82229	9.57222	9.35478	9.025	8.56818
4-1	$Al_2(So_4)_3$	4%	8.40751	8.21838	8.11841	7.89345	7.84669	7.84669	7.67942	7.18726	6.89344	6.68962	6.34414	5.67776
4-2			8.67841	8.44275	8.39	8.15149	8.11841	8.11841	7.9249	7.48979	7.17402	7.00583	6.65475	5.97086
4-3			8.58638	8.32058	8.20156	7.98847	7.87781	7.87781	7.67942	7.18726	6.86889	6.67796	6.33352	5.67776
5-1	P. granatum	4%	11.04003	10.89753	10.70364	10.54244	10.35992	10.13361	9.98771	9.528	8.94583	8.37254	7.84669	7.13458
5-2			10.48969	10.25821	10.06018	9.93993	9.72999	9.57222	9.39754	8.98526	8.4251	7.87781	7.36333	6.66634
5-3			10.48969	10.25821	10.15831	9.9162	9.75291	9.6618	9.39754	8.94583	8.4251	7.90914	7.41906	6.71304
6-1	C. sinensis	4%	9.22858	9.025	8.92624	8.75333	8.58638	8.47825	8.37254	7.98847	7.51841	7.08258	6.63168	6.00935
6-2			9.48415	9.29129	9.10545	8.96551	8.84863	8.64138	8.532	8.10196	7.62017	7.14768	6.66634	6.02876
6-3			9.20785	8.92624	8.84863	8.65986	8.51402	8.39	8.28626	7.89345	7.43311	7.00583	6.57463	5.94228
7-1	R. coriaria	4%	10.10902	9.93993	9.86904	9.6618	9.6618	9.68443	9.55007	9.16664	8.84863	8.58638	8.08557	7.32202
7-2			10.10902	9.84561	9.82229	9.6618	9.68443	9.61682	9.55007	9.1872	8.82942	8.58638	8.13492	7.32202
7-3			9.77594	9.59447	9.528	9.39754	9.41906	9.50603	9.44067	9.10545	8.84863	8.60464	8.20156	7.50408

8-1	Blank		0.15246	0.13584	0.12183	0.10857	0.09692	0.08875	0.08425	0.07944	0.07518	0.08875	0.08425	0.07944
8-2			0.14106	0.12552	0.11227	0.09875	0.08829	0.08078	0.07594	0.07113	0.06719	0.08078	0.07594	0.07113
8-3			0.14672	0.13113	0.11758	0.10424	0.09362	0.08539	0.07993	0.07506	0.07136	0.08539	0.07993	0.07506

No.	Mordant	RE	K/S Val(580)	K/S Val(590)	K/S Val(600)	K/S Val(610)	K/S Val(620)	K/S Val(630)	K/S Val(640)	K/S Val(650)	K/S Val(660)	K/S Val(670)	K/S Val(680)	K/S Val(690)	K/S Val(700)
1-1	-	2%	6.31237	5.92335	5.61695	5.35273	5.0873	4.84356	4.64998	4.51616	4.44009	4.37739	4.21898	4.10001	4.01559
1-2			6.56332	6.14753	5.81197	5.52363	5.22852	4.97335	4.72582	4.57011	4.49252	4.41712	4.2296	4.09496	3.99131
1-3			6.24966	5.84869	5.52363	5.24377	4.95941	4.71303	4.52806	4.38303	4.31615	4.25633	4.07488	3.95772	3.86886
2-1	-	4%	5.67776	5.22092	4.84356	4.49252	4.14075	3.7788	3.48128	3.22667	3.09426	2.99138	2.82868	2.61168	2.45685
2-2			5.38458	4.92485	4.546	4.19263	3.85056	3.51635	3.23696	2.99746	2.85953	2.76314	2.61414	2.4302	2.30128
2-3			5.66027	5.22092	4.86367	4.51616	4.1562	3.80545	3.5046	3.26466	3.12654	3.03125	2.86519	2.63643	2.4726
3-1	-	6%	8.88728	8.46046	8.15149	7.86222	7.60548	7.32202	7.08258	6.8445	6.76033	6.56332	6.39772	6.18803	6.00935
3-2			8.25219	7.84669	7.48979	7.16083	6.86889	6.58597	6.33352	6.14753	6.01904	5.90453	5.72189	5.5404	5.41675
3-3			8.16812	7.75465	7.3911	7.05683	6.73661	6.46307	6.19822	6.00935	5.8672	5.76663	5.5827	5.40062	5.25143
4-1	$Al_2(So_4)_3$	4%	5.05122	4.61899	4.25633	3.89656	3.52423	3.1298	2.80931	2.56083	2.4726	2.45015	2.27115	1.97992	1.8037
4-2			5.32906	4.8975	4.51616	4.11521	3.70919	3.28569	2.91698	2.63893	2.49536	2.428	2.24356	1.97505	1.81082
4-3			5.0584	4.61283	4.25633	3.89656	3.52817	3.14946	2.83146	2.61168	2.56083	2.56083	2.35284	2.0245	1.82374
5-1	P. granatum	4%	6.33352	5.5827	4.92485	4.32166	3.82339	3.4203	3.1168	2.90535	2.81483	2.76046	2.59213	2.34031	2.16928
5-2			5.90453	5.19071	4.51616	3.93874	3.46587	3.09106	2.81759	2.62154	2.52544	2.46583	2.32788	2.13259	1.99298
5-3			5.97086	5.24377	4.58834	4.01559	3.552	3.17598	2.89667	2.68705	2.58244	2.51149	2.36336	2.17486	2.0346
6-1	C. sinensis	4%	5.31338	4.66875	4.10001	3.60045	3.18601	2.84825	2.60432	2.42361	2.32581	2.25729	2.1344	1.97667	1.86629
6-2			5.33693	4.67503	4.07988	3.57206	3.16599	2.82868	2.58727	2.40833	2.32375	2.26124	2.13622	1.97182	1.86033
6-3			5.28225	4.64375	4.07988	3.5761	3.16931	2.84544	2.59699	2.41486	2.32581	2.25532	2.13259	1.97829	1.86778
7-1	R. coriaria	4%	6.43025	5.59978	4.87716	4.30517	3.86427	3.5046	3.22667	2.99746	2.85953	2.76314	2.59943	2.40833	2.26916
7-2			6.4521	5.60835	4.86367	4.28335	3.84147	3.48128	3.20624	2.98532	2.84544	2.74979	2.58727	2.39751	2.25532
7-3			6.68962	5.83947	5.09457	4.46909	4.01559	3.6375	3.36468	3.12329	2.97928	2.87372	2.70511	2.49536	2.34865

8-1	Blank	0.08875	0.08425	0.07944	0.07518	0.07086	0.05343	0.05211	0.05006	0.0485	0.04752	0.04605	0.04481	0.04336
8-2		0.08078	0.07594	0.07113	0.06719	0.06408	0.04752	0.04597	0.04453	0.0428	0.04221	0.04051	0.03919	0.03819
8-3		0.08539	0.07993	0.07506	0.07136	0.0673	0.04867	0.04722	0.04547	0.04424	0.04304	0.04198	0.04036	0.03971

No.	Mordant	RE	K/S Val(710)	K/S Val(720)	K/S Val(730)	K/S Val(740)	h (D65)	C* (D65)
1-1	-	2%	3.94347	3.93401	3.86427	3.8279	47.26	7.96
1-2			3.91054	3.89656	3.83241	3.79209	45.4	8.02
1-3			3.80099	3.78322	3.72208	3.6794	46.77	8.32
2-1	-	4%	2.36759	2.28915	2.2203	2.1435	41.71	11.28
2-2			2.22608	2.15633	2.10035	2.0346	41.1	11.99
2-3			2.38248	2.30331	2.23771	2.16002	42.24	11.21
3-1	-	6%	5.8672	5.77564	5.63421	5.51528	45.33	5.1
3-2			5.28225	5.22092	5.0873	4.98735	45.4	6.11
3-3			5.13856	5.07281	4.95941	4.87041	45.36	6.37
4-1	$Al_2(So_4)_3$	4%	1.72391	1.66034	1.61936	1.57039	41.94	13.41
4-2			1.73193	1.67049	1.62916	1.5786	40.72	13.01
4-3			1.73193	1.66034	1.61328	1.56223	43.26	13.49
5-1	P. granatum	4%	2.06709	1.97505	1.90873	1.82808	39.86	15.44
5-2			1.91027	1.83679	1.78117	1.71462	39.83	16.22
5-3			1.94781	1.87376	1.81368	1.74135	40.2	15.89
6-1	C. sinensis	4%	1.79803	1.73865	1.68847	1.6304	40.21	15.94
6-2			1.79098	1.72658	1.67816	1.62303	40.49	16.4
6-3			1.79803	1.73731	1.68588	1.62793	40.5	15.88
7-1	R. coriaria	4%	2.18046	2.10035	2.03799	1.96056	35.3	14.13
7-2			2.16928	2.09152	2.02786	1.95736	34.71	14.14
7-3			2.24943	2.16742	2.09328	2.0128	33	12.99

8-1	Blank	0.04269	0.04174	0.04097	0.04051	94.42	11.18
8-2		0.03724	0.03652	0.03588	0.03567	94.44	11.12
8-3		0.0387	0.03775	0.03731	0.0371	93.53	11.29

UV/ Visible spectrum

WL (nm)	Before dyeing		After dyeing	
	Abs	T%	Abs	T%
350	0.6266	23.63	0.3249	47.32
351	0.6175	24.13	0.3169	48.21
352	0.607	24.72	0.3103	48.94
353	0.598	25.24	0.3057	49.47
354	0.587	25.88	0.3035	49.71
355	0.5782	26.41	0.3016	49.94
356	0.568	27.04	0.2969	50.47
357	0.5586	27.63	0.2905	51.23
358	0.5488	28.26	0.2867	51.68
359	0.5382	28.96	0.282	52.24
360	0.5326	29.34	0.2773	52.8
361	0.5245	29.89	0.2728	53.36
362	0.5186	30.3	0.2706	53.62
363	0.5124	30.73	0.2668	54.1
364	0.5067	31.14	0.2632	54.55
365	0.5028	31.42	0.2591	55.06
366	0.4989	31.7	0.2553	55.55
367	0.4936	32.09	0.2514	56.05
368	0.4901	32.35	0.2483	56.46
369	0.4847	32.76	0.2449	56.89
370	0.4789	33.19	0.2432	57.12
371	0.4705	33.85	0.2382	57.78
372	0.4668	34.14	0.2361	58.06
373	0.4599	34.68	0.235	58.2
374	0.4571	34.9	0.232	58.62
375	0.4561	34.99	0.2278	59.19
376	0.4514	35.37	0.2252	59.54
377	0.4459	35.82	0.2225	59.9
378	0.4442	35.96	0.2194	60.33
379	0.4437	36	0.2175	60.6
380	0.4409	36.23	0.2156	60.88
381	0.4383	36.45	0.213	61.24
382	0.4328	36.92	0.2086	61.85
383	0.4356	36.68	0.2081	61.93
384	0.4337	36.84	0.2069	62.11
385	0.4321	36.98	0.2043	62.48
386	0.4313	37.05	0.2041	62.51
387	0.4322	36.97	0.202	62.81
388	0.4302	37.13	0.2007	63

389	0.4289	37.25	0.1992	63.21
390	0.431	37.07	0.1995	63.17
391	0.4303	37.13	0.1981	63.37
392	0.4294	37.2	0.1973	63.49
393	0.4295	37.2	0.1968	63.56
394	0.4307	37.09	0.1955	63.75
395	0.4311	37.06	0.1949	63.84
396	0.4324	36.95	0.194	63.98
397	0.4341	36.81	0.1945	63.89
398	0.4344	36.77	0.1936	64.03
399	0.4353	36.7	0.1931	64.11
400	0.4365	36.6	0.1926	64.18
401	0.4385	36.43	0.1928	64.15
402	0.4394	36.36	0.1927	64.17
403	0.4396	36.34	0.1919	64.28
404	0.4427	36.08	0.1923	64.23
405	0.4439	35.99	0.1917	64.31
406	0.4449	35.9	0.1927	64.17
407	0.4451	35.88	0.192	64.27
408	0.447	35.73	0.192	64.27
409	0.4476	35.68	0.1917	64.32
410	0.4478	35.66	0.1911	64.41
411	0.4502	35.47	0.1911	64.4
412	0.4515	35.36	0.1907	64.47
413	0.4522	35.3	0.1899	64.58
414	0.4531	35.23	0.1898	64.59
415	0.4537	35.18	0.1901	64.54
416	0.4546	35.1	0.1896	64.62
417	0.4553	35.05	0.1893	64.68
418	0.4559	35	0.1889	64.73
419	0.4556	35.02	0.1883	64.82
420	0.4564	34.96	0.1886	64.78
421	0.4581	34.83	0.1883	64.82
422	0.4591	34.75	0.1883	64.82
423	0.4591	34.75	0.1876	64.92
424	0.4596	34.71	0.1877	64.91
425	0.4598	34.69	0.187	65.01
426	0.4598	34.69	0.1873	64.97
427	0.46	34.67	0.1871	65
428	0.4592	34.74	0.1865	65.09
429	0.4589	34.76	0.1859	65.18
430	0.4586	34.79	0.1853	65.26
431	0.4579	34.84	0.1848	65.34
432	0.4573	34.89	0.1844	65.41
433	0.4567	34.94	0.1841	65.45
434	0.456	34.99	0.1836	65.52
435	0.4555	35.04	0.1831	65.6
436	0.455	35.07	0.1827	65.65
437	0.4535	35.19	0.1822	65.73
438	0.4526	35.27	0.1819	65.78

439	0.4521	35.31	0.1814	65.86
440	0.4503	35.46	0.1806	65.97
441	0.4494	35.53	0.1804	66.01
442	0.4473	35.7	0.1796	66.12
443	0.4461	35.8	0.1792	66.19
444	0.4445	35.93	0.1782	66.35
445	0.443	36.06	0.1773	66.48
446	0.4413	36.2	0.1761	66.66
447	0.4398	36.33	0.1757	66.72
448	0.4378	36.49	0.1747	66.88
449	0.4367	36.59	0.1743	66.94
450	0.4349	36.73	0.1741	66.97
451	0.4325	36.94	0.1723	67.25
452	0.43	37.15	0.1713	67.41
453	0.4275	37.37	0.1708	67.49
454	0.425	37.58	0.1699	67.62
455	0.4234	37.72	0.1682	67.89
456	0.4217	37.87	0.1681	67.9
457	0.4191	38.1	0.1669	68.09
458	0.4166	38.32	0.1666	68.13
459	0.4154	38.42	0.1659	68.25
460	0.4133	38.61	0.1652	68.35
461	0.4099	38.92	0.1644	68.48
462	0.4083	39.06	0.1635	68.63
463	0.4072	39.15	0.1627	68.75
464	0.405	39.35	0.1619	68.88
465	0.4022	39.61	0.1607	69.07
466	0.3995	39.86	0.1603	69.14
467	0.3983	39.97	0.1593	69.29
468	0.3973	40.06	0.1584	69.43
469	0.3948	40.29	0.1578	69.54
470	0.3936	40.4	0.1574	69.59
471	0.3916	40.59	0.1566	69.72
472	0.3888	40.85	0.1556	69.88
473	0.3867	41.05	0.1546	70.05
474	0.3851	41.2	0.1538	70.18
475	0.3832	41.38	0.1531	70.29
476	0.3812	41.57	0.152	70.46
477	0.3792	41.77	0.1517	70.52
478	0.3781	41.87	0.1506	70.7
479	0.3767	42.01	0.1501	70.78
480	0.3746	42.21	0.1492	70.93
481	0.3726	42.4	0.1482	71.09
482	0.3717	42.49	0.147	71.28
483	0.3694	42.72	0.1468	71.31
484	0.3672	42.93	0.1462	71.41
485	0.3654	43.11	0.1451	71.59
486	0.3649	43.17	0.144	71.78
487	0.363	43.35	0.1432	71.92
488	0.3618	43.47	0.1425	72.02

489	0.3594	43.71	0.1421	72.09
490	0.3577	43.89	0.1415	72.19
491	0.3552	44.13	0.1401	72.42
492	0.3537	44.29	0.1396	72.52
493	0.3518	44.48	0.1386	72.68
494	0.3517	44.5	0.1384	72.7
495	0.3499	44.67	0.1375	72.86
496	0.3475	44.93	0.1364	73.05
497	0.3457	45.11	0.1357	73.17
498	0.3439	45.3	0.1348	73.31
499	0.3423	45.47	0.134	73.45
500	0.3402	45.69	0.1335	73.54
501	0.3387	45.85	0.1325	73.71
502	0.337	46.03	0.1316	73.86
503	0.3353	46.21	0.1309	73.98
504	0.3333	46.42	0.13	74.13
505	0.331	46.66	0.1295	74.21
506	0.3294	46.84	0.1288	74.34
507	0.3274	47.05	0.1278	74.5
508	0.3263	47.17	0.1271	74.63
509	0.3237	47.46	0.1259	74.83
510	0.3215	47.7	0.1257	74.87
511	0.319	47.97	0.1249	75.01
512	0.3165	48.25	0.1239	75.18
513	0.3142	48.51	0.1229	75.35
514	0.3122	48.73	0.1222	75.47
515	0.3098	49	0.1213	75.64
516	0.3076	49.25	0.1206	75.75
517	0.3051	49.54	0.1198	75.9
518	0.3027	49.81	0.1188	76.07
519	0.3008	50.02	0.1182	76.17
520	0.2983	50.32	0.1173	76.34
521	0.2953	50.66	0.1165	76.46
522	0.293	50.93	0.1156	76.63
523	0.2903	51.25	0.1148	76.77
524	0.2874	51.59	0.1139	76.93
525	0.2853	51.84	0.1131	77.07
526	0.2824	52.19	0.1124	77.2
527	0.2799	52.49	0.1116	77.35
528	0.2773	52.81	0.1107	77.5
529	0.2752	53.06	0.11	77.62
530	0.2727	53.37	0.1092	77.77
531	0.2698	53.72	0.1083	77.93
532	0.2676	54	0.1077	78.03
533	0.2652	54.3	0.107	78.16
534	0.2624	54.65	0.1062	78.31
535	0.2601	54.94	0.1052	78.48
536	0.2578	55.24	0.1046	78.6
537	0.2554	55.54	0.1035	78.8
538	0.2518	56	0.1024	78.99

539	0.2493	56.33	0.102	79.07
540	0.2473	56.59	0.1014	79.18
541	0.2454	56.84	0.1018	79.1
542	0.2424	57.23	0.1006	79.32
543	0.2403	57.5	0.1	79.43
544	0.2381	57.8	0.0996	79.51
545	0.2357	58.12	0.0988	79.65
546	0.2338	58.38	0.0982	79.77
547	0.2308	58.78	0.0973	79.94
548	0.2283	59.12	0.097	79.99
549	0.2259	59.45	0.0963	80.1
550	0.2229	59.86	0.0955	80.26
551	0.2204	60.21	0.0949	80.36
552	0.2182	60.5	0.0946	80.42
553	0.216	60.81	0.0941	80.53
554	0.2139	61.11	0.0932	80.68
555	0.2119	61.4	0.0929	80.73
556	0.2093	61.77	0.0922	80.88
557	0.2068	62.12	0.0918	80.94
558	0.2046	62.43	0.0913	81.03
559	0.202	62.8	0.0907	81.16
560	0.1997	63.14	0.0901	81.27
561	0.1971	63.53	0.0894	81.4
562	0.1942	63.94	0.0886	81.55
563	0.1914	64.36	0.0882	81.62
564	0.1894	64.66	0.0875	81.74
565	0.1876	64.92	0.0874	81.77
566	0.1856	65.22	0.0872	81.81
567	0.1834	65.55	0.0865	81.95
568	0.1817	65.82	0.0858	82.07
569	0.1791	66.21	0.0853	82.16
570	0.177	66.52	0.0848	82.25
571	0.1752	66.8	0.0843	82.36
572	0.1732	67.11	0.0839	82.44
573	0.171	67.46	0.0833	82.55
574	0.1688	67.79	0.0827	82.65
575	0.1668	68.1	0.0824	82.73
576	0.1652	68.36	0.0822	82.76
577	0.1639	68.56	0.0823	82.74
578	0.1623	68.81	0.0818	82.84
579	0.1608	69.05	0.0813	82.92
580	0.1591	69.33	0.081	82.98
581	0.1579	69.52	0.0808	83.02
582	0.1566	69.73	0.0803	83.11
583	0.1549	70	0.08	83.18
584	0.1531	70.3	0.0798	83.22
585	0.1518	70.5	0.0794	83.3
586	0.1495	70.88	0.0756	84.03
587	0.148	71.12	0.0752	84.11
588	0.1469	71.3	0.0745	84.23

589	0.1458	71.49	0.0742	84.29
590	0.1439	71.8	0.074	84.34
591	0.1427	72	0.074	84.32
592	0.1417	72.16	0.0737	84.4
593	0.1404	72.37	0.0731	84.5
594	0.1389	72.62	0.073	84.52
595	0.1384	72.71	0.0729	84.55
596	0.1373	72.89	0.0727	84.6
597	0.1362	73.07	0.0721	84.71
598	0.1343	73.4	0.0718	84.77
599	0.1325	73.71	0.0717	84.79
600	0.1319	73.81	0.0714	84.83
